# Long-Term Safety and Feasibility of Right Atrial Septal Pacing with Lumenless Leads in Patients with Sick Sinus Syndrome

**DOI:** 10.1016/j.cjco.2025.06.014

**Published:** 2025-06-24

**Authors:** Peng Jin, Lan Su, Tiantian Chen, Shenglong Zheng, Zhongping Yang, Hao Zhou, Xiao Chen, Shengjie Wu, Lu Lin, Xi Zhou, Xue Xia, Weijian Huang

**Affiliations:** a Department of Cardiology, First Affiliated Hospital of Wenzhou Medical University, Wenzhou, China; b The Key Lab of Cardiovascular Disease, Science and Technology of Wenzhou, Wenzhou, China; c Cardiac Rhythm Management, Medtronic, Mounds View, Minnesota, USA

**Keywords:** Sick sinus syndrome, atrial fibrillation, atrial septal pacing, right atrial appendage pacing

## Abstract

**Background:**

Right atrial appendage pacing (RAAp) may increase the risk of atrial fibrillation (AF), compared to right atrial septal pacing (RASp). However, the implantation of atrial septal stylet-driven leads (SDLs) for RASp can present procedural challenges and limit its clinical application. We evaluated the long-term safety and feasibility of using lumenless leads (LLLs) with the delivery sheath for RASp and SDLs for RAAp, and compared AF events between the RASp and RAAp in patients with sick sinus syndrome.

**Methods:**

A total of 329 patients with sick sinus syndrome who underwent pacemaker implantation were divided into 2 groups, based on the site of atrial lead placement: the RASp group (n = 162) with LLLs, and the RAAp group (n = 167) with SDLs. Implantation success rate, procedural time, P-wave characteristics, pacing parameters, complications, and AF episodes were compared between the 2 groups.

**Results:**

The success rates were similar for the RASp and RAAp groups (98.8% vs 97.6%, *P* > 0.05). The lead implantation time was significantly shorter in the RASp group (2.5 ± 1.9 minutes vs 10.3 ± 2.9 minutes, *P* < 0.05). During a mean follow-up of 36.4 ± 20.5 months, the pacing parameters remained stable without serious complications. Additionally, the RASp group had a significantly reduced incidence of AF episodes (6.7% vs 14.0%, *P* < 0.05) and new-onset AF (1.8% vs 4.6%, *P* < 0.05).

**Conclusions:**

The long-term safety and feasibility of RASp with LLLs were comparable to those of RAAp patients with SDLs. The RASp reduced the incidence of postoperative AF episodes and new-onset AF. The RASp by delivery sheath implantation is a safe and effective method.

## Résumé

**Contexte:**

La stimulation de l’appendice auriculaire droit (SAAD) peut augmenter le risque de fibrillation auriculaire (FA), comparativement à la stimulation du septum auriculaire droit (SSAD). Cependant, l’implantation d’une sonde à stylet interauriculaire (SSIA) dans le cadre de la SSAD peut entraîner des difficultés procédurales et limiter son utilisation dans la pratique clinique. Nous avons évalué l’innocuité à long terme et la possibilité d’utiliser une sonde sans lumière (SSL) avec la gaine d’introduction pour la SSAD et une SSIA pour la SAAD, et comparé les épisodes de FA entre la SSAD et la SAAD chez des patients atteints d’une dysfonction du nœud sinusal.

**Méthodologie:**

Au total, 329 patients atteints d’une dysfonction du nœud sinusal chez qui un stimulateur cardiaque a été implanté ont été répartis dans deux groupes selon le site d’implantation de la sonde auriculaire : le groupe SSAD avec SSL (n = 162) et le groupe SAAD avec SSIA (n = 167). Le taux de réussite de l’implantation, la durée de l’intervention, les caractéristiques de l’onde P, les paramètres de stimulation, les complications et les épisodes de FA ont été comparés entre les deux groupes.

**Résultats:**

Le taux de réussite a été similaire entre le groupe SSAD et le groupe SAAD (98,8 % *vs* 97,6 %; *p* > 0,05). Le temps d’implantation de la sonde a été significativement plus court dans le groupe SSAD (2,5 ± 1,9 minutes vs 10,3 ± 2,9 minutes; *p* < 0,05). Durant un suivi moyen de 36,4 ± 20,5 mois, les paramètres de stimulation sont demeurés stables, sans qu’aucune complication grave soit observée. En outre, l’incidence des épisodes de FA a été nettement moindre dans le groupe SSAD que dans le groupe SAAD (6,7 % vs 14,0 %; *p* < 0,05), toute comme celle de FA d’apparition nouvelle (1,8 % vs 4,6 %; *p* < 0,05).

**Conclusions:**

L’innocuité à long terme et la possibilité d’utiliser une SSAD avec une SSL ont été comparables à celles des patients recevant une SAAD avec SSIA. La SSAD a réduit l’incidence des épisodes de FA postopératoires ainsi que celle des épisodes de FA d’apparition nouvelle. La SSAD avec implantation à l’aide d’une gaine d’introduction est une méthode sûre et efficace.

Pacemaker therapy is the primary treatment for patients with sick sinus syndrome (SSS)-related bradycardia.^1^ Lamas et al. collected data on patients with SSS in the Mode Selection Trial (MOST) and found that up to 46% of patients had a history of atrial fibrillation (AF).^2^ The bradycardia induced by SSS may promote the occurrence of AF by increasing atrial ectopic beats and dispersion of atrial refractoriness (Central Illustration).^3^

The right atrial appendage is the conventional site for implanting an atrial pacing lead, due to its ease of fixation. But its thin myocardial wall makes it prone to perforation, which may lead to pericarditis or effusion.^4^ In addition, researches have shown that right atrial appendage pacing (RAAp) can lead to prolonged P-wave duration, increased P-wave dispersion, and a longer PR interval.^5^, ^6^, ^7^ Alternatively, using the right atrial septum for an atrial pacing lead implant site may reduce conduction anisotropy in the atrium, improve inter-atrial synchronization, lower the incidence of AF, and enhance left ventricular diastolic function.^8^, ^9^, ^10^

The 2 primary pacing sites within the right atrial septum are as follows: (i) the high atrial septum including the Bachmann's bundle area; and (ii) the low atrial septum.^9^ Several short-term studies have shown that using lumenless leads (LLLs) with an implant sheath can significantly improve the success rate of implantation, increasing it from 54% to 88.9%.^4^^,^^11^, ^12^, ^13^, ^14^ In contrast, conventional stylet-driven leads (SDLs) exhibit a much lower success rate of only 21%. Moreover, SDLs carry the additional risk of being implanted near the aortic sinus,^12^ which potentially could lead to aortic sinus perforation.

This study reviewed our single-centre clinical experience, which demonstrated high implant success rates with right atrial septal pacing (RASp) using LLLs in patients with SSS. Our study emphasized the long-term safety, lead performance, and clinical outcome of suppressing AF.

## Methods

### Study population

This single-centre, retrospective study included 329 consecutive patients with SSS admitted between February 2016 and June 2023 at the First Affiliated Hospital of Wenzhou Medical University (Wenzhou, China). Patients were divided into 2 groups based on the site of final atrial lead placement—the RASp group (n = 162) with LLLs, and the RAAp group (n = 167) with SDLs. The inclusion criteria were as follows: (i) indication for implantation of dual-chamber pacemakers in patients with SSS; (ii) the ability to complete data collection; and (iii) clinically significant sinus bradycardia, sinus arrest, or tachy-brady syndrome with associated symptoms. We excluded patients who had a life expectancy of < 1 year, had a history of longstanding persistent AF, or had a recent history of AF ablation. All patients who underwent atrial pacing were followed up for a minimum of 1 year after the procedure. This study was approved by the Ethics Committee of the first affiliated hospital of Wenzhou Medical University.

In the RAAp group, 163 of 167 SDLs (97.6%) were implanted successfully, compared to 160 of 162 LLL implants (98.8%) in the RASp group. In the successful RASp group, the atrial septum pacing site of the atrial septum was determined by combining fluoroscopy and paced P-wave morphology. The Medtronic SelectSecure 3830TM (Medtronic, Minneapolis, MN) was the predominant lead used for RASp, and the conventional SDL was commonly used for RAA lead implantation at our institution. The atrial lead and ventricular lead models are shown in Table 1.

**Table 1:** Characteristics

Category	RASp (n = 162)	RAAp (n = 167)	*P*
Sex, n (% male)	63 (38.9)	80 (47.9)	0.078
Age, y	67.9 ± 8.9	67.6 ± 9.8	0.793
Smoking history	24 (14.8)	26 (15.6)	0.850
Drinking history	25 (15.4)	20 (12.0)	0.363
Underlying disease			
Hypertension	88 (54.3)	83 (49.7)	0.403
Diabetes	20 (12.3)	16 (9.6)	0.423
Coronary artery disease	25 (15.4)	24 (14.4)	0.788
Cardiomyopathies			
DCM	2 (1.2)	0 (0.0)	—
HCM	0 (0.0)	2 (1.2)	—
ACM	2 (1.2)	0 (0.0)	—
ICM	0 (0.0)	0 (0.0)	—
Valvular disease	1 (0.6)	3 (1.8)	0.628
Renal dysfunction (eGFR <30 mL/[min · 1.73 m^2^]),	7 (4.3)	4 (2.4)	0.333
NYHA functional class III/IV	11 (6.8)	8 (4.8)	0.438
NYHA functional class II	9 (5.6)	12 (7.2)	0.547
Medications			
ACEI/ARB	45 (27.8)	48 (28.7)	0.847
Beta-blocker	29 (17.9)	42 (25.1)	0.111
Amiodarone	10 (6.2)	16 (9.6)	0.253
NDHP-CCB	1 (0.6)	0 (0.0)	—
DHP-CCB	50 (30.9)	50 (29.9)	0.856
Atrial lead			
Model 3830, Medtronic	152 (93.8)	5 (3.0)	0.001
Model 5076, Medtronic	5 (3.1)	125 (74.9)	0.001
Model 2088TC, Abbott	4 (2.5)	29 (17.4)	0.001
Model Solia S, Biotronik	1 (0.6)	0 (0.0)	—
Model 7741, Boston Scientific	0 (0.0)	8 (4.8)	—
Ventricular lead			
Model 3830, Medtronic	162 (100)	167 (100)	—
%VP, %	14.1	15.1	0.771
%AP, %	61.7	59.8	0.561

Values are given as mean ± SD or n (%), unless otherwise indicated. Numerical variables were tested using the *t*-test. Categorical variables were evaluated using the χ^2^ or Fisher exact tests. Company locations are as follows: Abbott—Abbott Park, IL; Biotronik—Berlin, Germany; Boston Scientific—Boston, MA; Medtronic—Minneapolis, MN.

ACEI, angiotensin-converting enzyme inhibitor; ACM, atrial cardiomyopathy; ARB, angiotensin receptor blocker; DCM, dilated cardiomyopathy; DHP-CCB, dihydropyridine-calcium channel blocker; eGFR, estimated glomerular filtration rate; HCM, hypertrophic cardiomyopathy; ICM, Ischemic cardiomyopathy; NDHP-CCB, non-dihydropyridine-calcium channel blocker; NYHA, New York Heart Association; RAAp, right atrial appendage pacing; RASp, right atrial septal pacing %AP, atrial percentage; %VP, ventricular percentage.

### Implantation procedure

#### Preoperative preparation

Preoperative baseline data of enrolled patients were collected, and contraindications to surgery were ruled out by routine preoperative evaluations, including a 12-lead electrocardiogram (ECG), echocardiography, and hematological tests. ECG and arrhythmia data collection encompassed P-wave duration, P-wave dispersion, and AF-related data (recorded via Holter ECG or continuous ECG monitoring). Standard echocardiographic indices, such as left atrial diameter (LAD), left ventricular end-diastolic dimension (LVEDD), and left ventricular ejection fraction, also were recorded.

#### Device implantation

**Figure 1:**
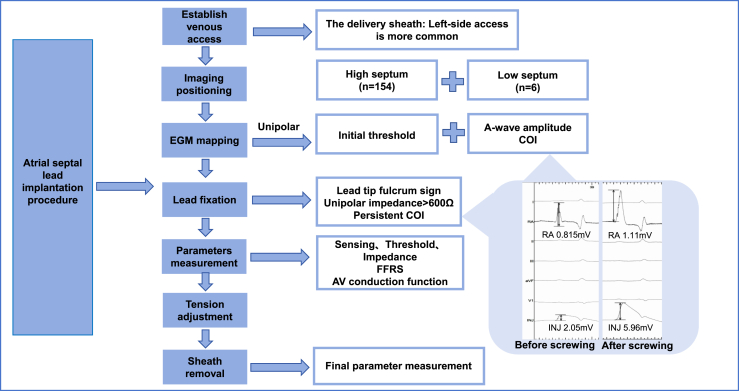
Atrial septal lead implantation procedure. AV, atrioventricular; COI, current of injury; EGM, electrogram; FFRS, far field R-wave sensing; INJ, injury; RA, right atrium.

**Figure 2:**
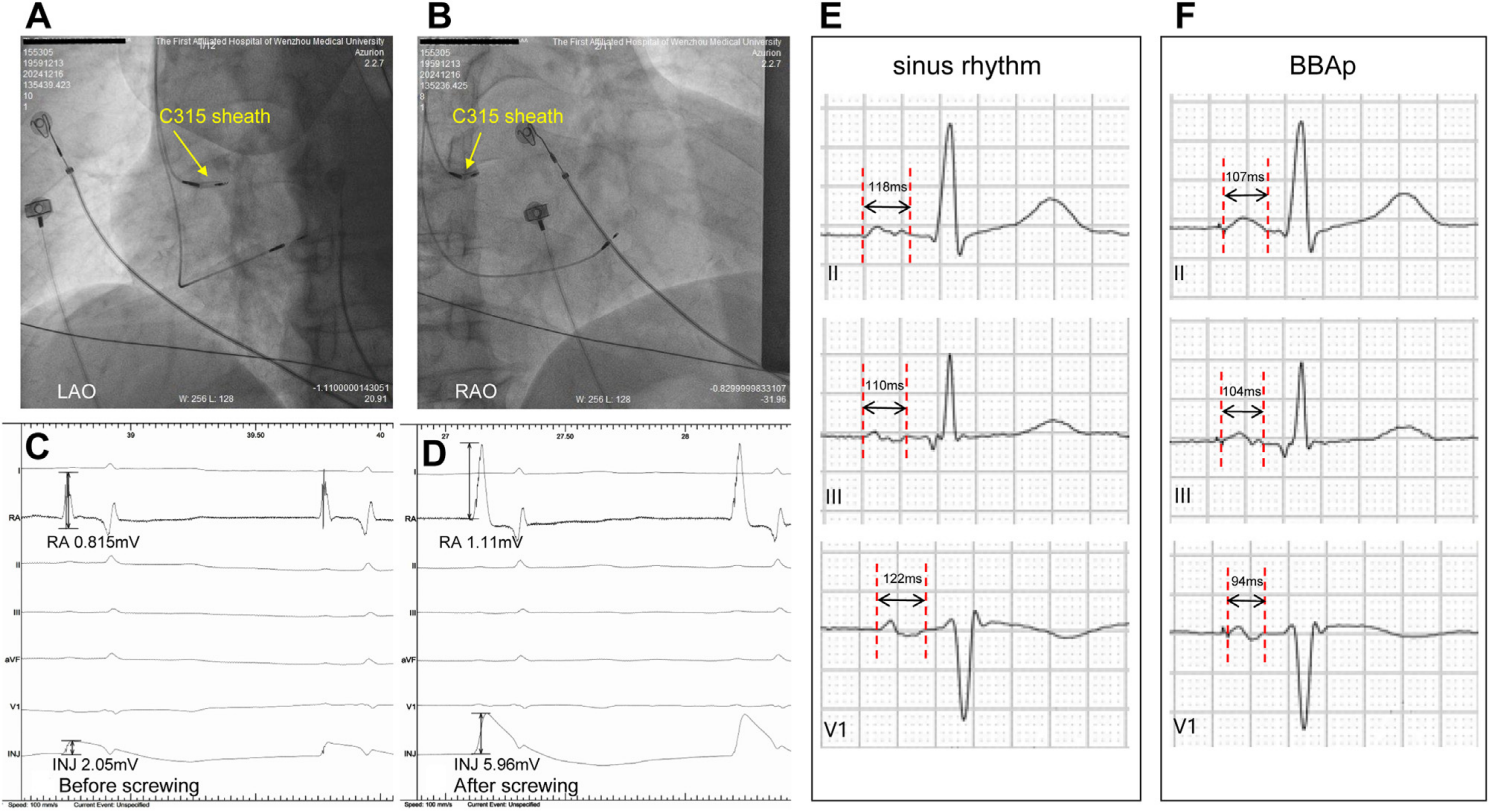
Fluoroscopy and P-wave characteristics for right atrial septal pacing. (**A,B**) Fluoroscopy for right atrial septal lead with the delivery C315 sheath. (**C,D**) Electrogram of atrial wave injury currents before and after screwing during implantation; speed, 100 mm/s. (**E,F**) Electrocardiograms are shown at 50 mm/s during sinus rhythm and right atrial septal pacing. BBAp, Bachmann’s bundle area pacing. INJ, injury; LAO, left anterior oblique; RA, right atrium; RAO, right anterior oblique; RASp, right atrial septal pacing.

For patients in the RASp group, the lumenless SelectSecure lead (Model 3830, Medtronic) and delivery sheath (C315HIS, Medtronic) were used (Fig. 1). During the RASp implantation procedure, atrial sensing and pacing thresholds were assessed using the EP recording system and a pacing system analyzer, alongside a 12-lead ECG. Atrial lead positioning was guided by fluoroscopy and atrial wave mapping. Atrial wave potential mapping was performed using a dual-channel recording method using the EP recording system, which allowed simultaneous display of the atrial wave amplitude and current of injury (COI; Fig. 2). Unipolar mapping facilitated intraoperative measurements.

Under the fluoroscopic left anterior oblique (LAO) view, the lead with the delivery sheath was delivered from the superior vena cava to the top of the roof of the right atrium; the delivery sheath was rotated carefully clockwise to align perpendicularly with the right atrial septum, avoiding the foramen ovale. The posterior septal position was confirmed in the right anterior oblique view to prevent orientation toward the aortic sinus. The lead body was screwed for 4-6 turns, with the isotropic COI ensuring secure fixation. After fixation, initial atrial sensing, pacing thresholds, and impedance were tested, with additional turns made if necessary. The total LLL implantation time was defined as the time from delivery sheath advancement into the right atrium to achievement of satisfactory parameters after lead tension adjustment prior to sheath cutting.

Stylet-driven leads were used for the RAAp implant, with fixation in the RAA confirmed under fluoroscopy. The lead tip was confirmed to be away from the spine in the LAO view. The stability of the fixation was then assessed using the isotropic COI, followed by testing of the initial pacing parameters. Once these parameters were deemed satisfactory, the J-shaped stylet was removed. The SDL implantation time was defined as the duration from when the lead passed through the introducer sheath to when the parameters were satisfactory and the J-shaped stylet was exchanged for a straight stylet.

### Data collection and follow-up

Before implantation, 290 patients (89.8%) underwent Holter ECG monitoring, and 33 patients (10.2%) underwent continuous ECG monitoring, which identified AF events in 113 patients. Among these, 53 patients (33.1%) were in the RASp group, and 60 patients (36.8%) were in the RAAp group.

Standard 12-lead ECGs were used to measure the P-wave duration and dispersion. P-wave parameters were assessed by marking the onset when the trace left the isoelectric line and the end when it returned. P-wave duration was measured from start to end, and paced P-wave duration was calculated from the end of the pacing spike to the P-wave's end. P-wave dispersion (Pd) was defined as the difference between the maximum and minimum P-wave durations observed.^15^

During the implantation procedure, parameters such as atrial sensing, pacing thresholds, and impedance were recorded for both unipolar and bipolar modes, and COIs also were recorded. The total procedure time (from administration of local anesthesia to pocket closure) and the atrial lead implantation time were documented.

Post implantation, patients attended regular follow-ups. Pacemaker programming data monitored AF burden or AF events. We defined AF events as an atrial rate of 350 beats per minut,e lasting > 6 seconds, based on the following rationale:

1. Postoperative AF events were detected with high sensitivity via pacemaker recordings. To avoid misclassification of false-positive AF events, atrial sensing was set to bipolar mode in all cases postoperatively, thereby reducing interference caused by unipolar sensing or double counting due to far-field ventricular signals.
2. Additionally, only events with an atrial rate of > 350 beats per minute were defined as AF, confirmed as true events by reviewing the list of events and intracardiac electrograms, while excluding other atrial arrhythmias such as atrial tachycardia.

New-onset AF events were defined as those that were undetected preoperatively but met the above AF events criteria postoperatively. AF burden was determined using pacemaker counters, with corresponding data provided in the programming report. Complications such as pocket hematoma, pocket infection, lead dislodgement, or loss of capture were also tracked.

### Statistical analysis

Quantitative data were expressed as mean ± standard deviation ($\bar{x}\pm$ s) or median (interquartile range). An independent sample *t*-test or Wilcoxon rank-sum test was used for comparison between the groups, and a paired sample *t*-test or Wilcoxon signed-rank test was used for comparisons within the group. Qualitative data were expressed as percentage, and comparisons were made using the χ^2^ test or Fisher's exact test. Survival from AF was estimated using Kaplan-Meier methods, and relative risk and 95% confidence intervals were calculated by Cox proportional hazards methods. A value of *P* < 0.05 was considered statistically significant. Statistical analysis was performed using SPSS 26.0 software (IBM, Armonk, NY).

## Results

### Baseline characteristics and implantation success

Among the 329 patients, a total of 6 cases with failed implants were recorded and excluded from the statistical analysis. The total success rate was 98.2%, and the success rate was similar for the RASp and RAAp groups (98.8% vs 97.6%, *P* > 0.05). The failed cases included 2 cases with LLLs, due to poor parameters. Four cases with SDLs failed to achieve fixation in the right atrial appendage and were switched to 3830 lead implantation at the atrial septum. Baseline characteristics for both the RASp and RAAp groups are summarized in Table 1.

### Total procedural and atrial lead procedural time

The total procedural time was 83.1 ± 31.6 minutes for the RASp group, and 76.5 ± 29.3 minutes for the RAAp group, with no statistically significant difference (*P* > 0.05). However, the 2 groups did have a statistically significant difference in atrial lead procedural time: 2.5 ± 1.9 minutes for the RASp group vs 10.3 ± 2.9 minutes for the RAAp group (*P* < 0.05).

### Atrial lead electrical performance

**Figure 3:**
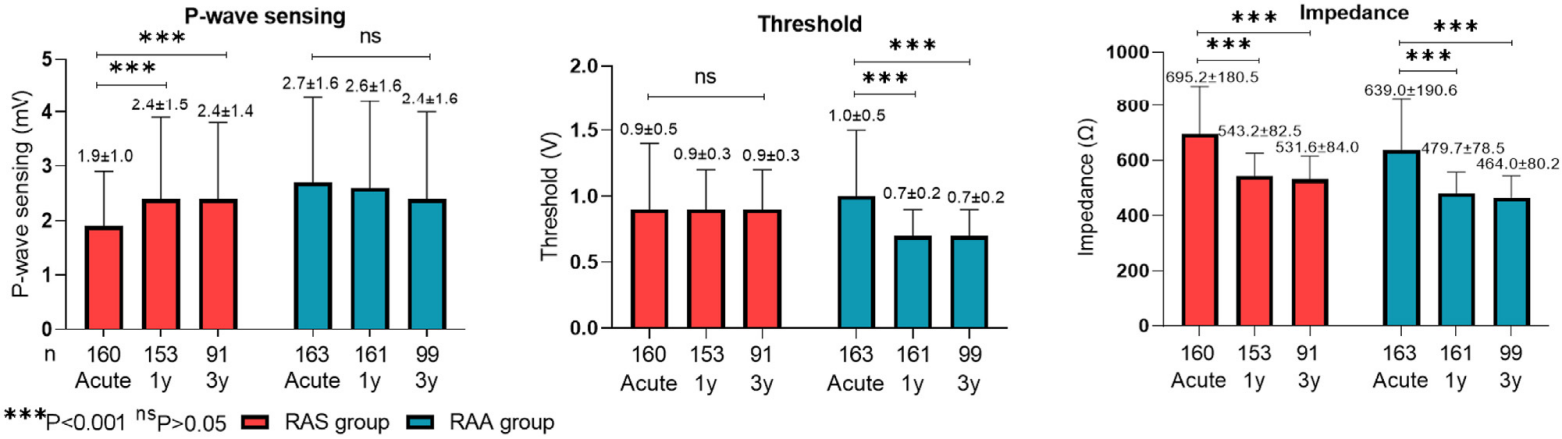
Atrial lead electrical performance. Shown is the mean ± standard deviation bipolar atrial lead electrical performance in both groups. The postoperative parameters measurement used bipolar mapping. ns, nonsignificant; RAA, right atrial appendage; RAS, right atrial septal.

Postoperative electrical performance of the atrial leads was followed up over an average of 36.4 ± 20.5 months. In the RASp group, a significant increase occurred in the atrial bipolar sensing at the 1-year follow-up (2.4 ± 1.5 mV vs 1.9 ± 1.0 mV, *P* < 0.001), after which it gradually stabilized. Conversely, atrial bipolar sensing in the RAAp group did not show significant changes over time. In the RAAp group, the postoperative bipolar pacing threshold significantly decreased at the 1-year follow-up (0.7 ± 0.2 V vs 1.0 ± 0.5 V, *P* < 0.001), whereas the bipolar pacing threshold in the RASp group remained stable over time. In addition, at the 1-year follow-up, the postoperative bipolar pacing impedance showed a decrease in both the RASp group (543.2 ± 82.5 Ω vs 695.2 ± 180.5 Ω at the implant, *P* < 0.001) and the RAAp group (479.7 ± 78.5 Ω vs 639.0 ± 190.6Ω at the implant, *P* < 0.001). This trend continued until the impedance gradually stabilized, as illustrated in Figure 3.

### Lead-related complications

The mean follow-up period was 36.4 ± 20.5 months. One case of poor atrial sensing (< 0.2 mV) occurred in the RAAp group. No patients experienced lead-related complications, such as pocket hematoma, pocket infection, lead dislodgment, loss of capture, or significant pericardial effusion.

### P-wave duration and P-wave dispersion

The mean P-wave duration during RASp was significantly shorter than that during both sinus rhythm (103.7 ± 13.3 ms vs 128.0 ± 15.0 ms, *P* < 0.05) and RAAp (103.7 ± 13.3 ms vs 128.0 ± 12.5 ms, *P* < 0.05). The mean P-wave duration during RAAp was longer than that during sinus rhythm (128.0 ± 12.5 ms vs 124.3 ± 12.7 ms, *P* < 0.05). The mean P-wave dispersion during RASp was shorter than that during sinus rhythm (17.4 ± 6.8 ms vs 22.7 ± 8.0 ms, *P* < 0.05), whereas the mean P-wave dispersion during RAAp showed no significant change (17.4 ± 6.8 ms vs 17.5 ± 6.5 ms, *P* > 0.05; Table 2).

**Table 2:** P-wave duration and P-wave dispersion

Group	RASp (n = 160)	RAAp (n = 163)	HASp (n = 115)	BBAp (n = 39)	*P*
Sinus P-wave duration, ms	128.0 ± 15.0	124.3 ± 12.7	127.5 ± 12.8	129.3 ± 19.8	*P*1 = 0.051 *P*2 = 0.425
Sinus P-wave dispersion, ms	22.7 ± 8.0	17.5 ± 6.6	22.4 ± 8.1	23.3 ± 7.8	*P*1 = 0.000 *P*2 = 0.702
Paced P-wave duration, ms	103.7 ± 13.3∗	128.0 ± 12.5∗	102.2 ± 12.5∗	107.6 ± 14.9∗	*P*1 = 0.000 *P*2 = 0.614
Paced P-wave dispersion, ms	17.4 ± 6.8∗	17.5 ± 6.5†	16.8 ± 6.2∗	19.1 ± 8.3∗	*P*1 = 0.916 *P*2 = 0.133

Values are given as mean ± standard deviation, unless otherwise indicated. Numerical variables were tested using the *t*-test. *P*1 indicates right atrial septal pacing (RASp) vs right atrial appendage pacing (RAAp); *P*2 indicates high atrial septal pacing (HASp) vs Bachmann’s bundle area pacing (BBAp).

∗,^†^Compared with this group before pacing.

∗ *P* < 0.05.

† *P* > 0.05.

### The occurrence of postoperative AF events

**Figure 4:**
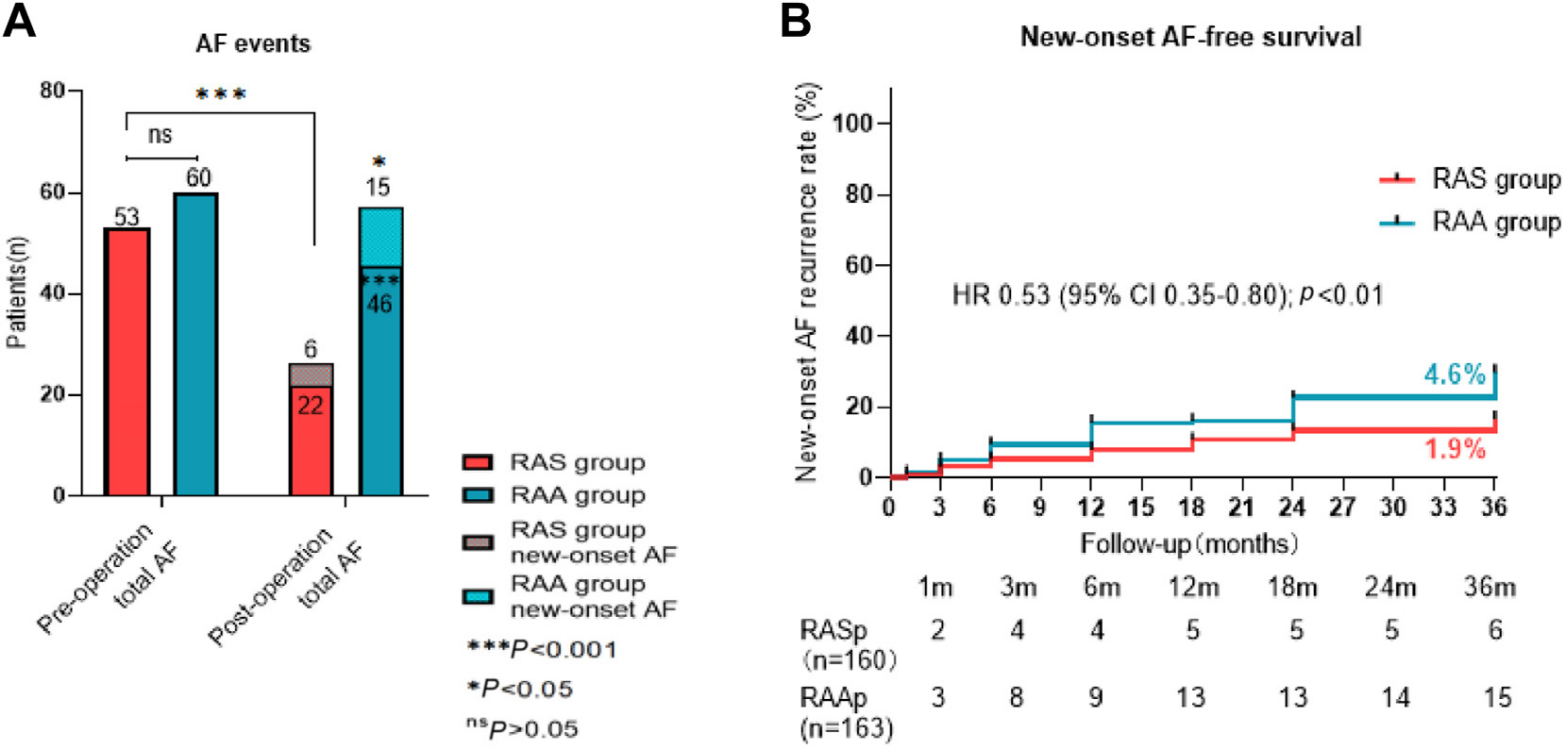
The occurrence of atrial fibrillation (AF) events. (**A**) Shown are the total AF events and new-onset AF events in patients with right atrial septum (RAS), and right atrial appendage (RAA) pacing. (**B**) Kaplan-Meier curve shows survival free from new-onset AF in both groups. CI, confidence interval; HR, hazard ratio; ns, nonsignificant; RASp, RAS pacing; RAAp, RAA pacing.

Figure 4 shows that the incidence of AF events decreased in both groups, with the total incidence being lower in the RASp group vs that in the RAAp group (6.7% vs 14.0%, *P* = 0.001), and the incidence of new-onset AF events was lower in the RASp group (n = 6) than in the RAAp group (n = 15, 1.8% vs 4.6%, *P* = 0.047) during the mean follow-up of 36.4 ± 20.5 months. In terms of the postoperative AF burden, the RASp (n = 21) and RAAp (n = 27) groups had a burden of < 40% (*P* = 0.385), and 8 patients in each group had a burden of ≥ 40% (*P* = 0.970). The Kaplan-Meier curve demonstrated that the risk of new-onset AF occurrence was significantly lower in RASp patients vs that in RAAp patients (hazard ratio 0.53; 95% CI 0.35, 0.80; *P* < 0.01; Fig. 4).

### Cardiac function and echocardiographic parameters

During postoperative follow-up, patients with a NYHA functional class of II or higher in both groups showed improvement in cardiac function (*P* < 0.05 for both groups). In the RASp group, the number of patients with a NYHA functional class of II or higher decreased from 20 to 4, and in the RAAp group, it decreased from 20 to 6. At the 1-year follow-up, both groups had a postoperative left ventricular ejection fraction > 50%. In the RASp group, the postoperative LAD (43.5 ± 5.6 mm vs 42.2 ± 6.0 mm, *P* < 0.05) and LVEDD (49.8 ± 5.1 mm vs 47.5 ± 4.5 mm, *P* < 0.05) were reduced, compared to the preoperative LAD. Similar results were observed in the RAAp group, in which postoperative LAD (42.2 ± 4.6 mm vs 41.1 ± 6.1 mm, *P* < 0.05) and LVEDD (49.6 ± 5.1 mm vs 47.8 ± 4.5 mm, *P* < 0.05) were also reduced (Table 3).

**Table 3:** Echocardiographic parameters

		LAD, mm		LVEDD, mm		LVEF, %	
Group	n	Baseline	Post-pacing (1 y)	Baseline	Post-pacing (1 y)	Baseline	Post-pacing (1 y)

RASp	160	43.5 ± 5.6 (n = 160)	42.2 ± 6.0∗ (n = 160)	49.8 ± 5.1 (n = 160)	47.5 ± 4.5∗ (n = 160)	68.0 ± 7.6 (n = 160)	65.2 ± 4.7∗ (n = 160)
RAAp	163	42.2 ± 4.6 (n = 163)	41.1 ± 6.1∗ (n = 163)	49.6 ± 5.1 (n = 163)	47.8 ± 4.5∗ (n = 163)	67.7 ± 6.6 (n = 163)	65.0 ± 5.7∗ (n = 163)
*P*		0.023	0.109	0.661	0.513	0.703	0.674

Values are given as mean ± standard deviation, unless otherwise indicated. Numerical variables were tested using the *t*-test.

LAD, left atrial diameter; LVEDD, left ventricular end-diastolic dimension; LVEF, left ventricular ejection fraction; RAAp, right atrial appendage pacing; RASp, right atrial septal. pacing.

∗ Compared with this group before pacing, *P* < 0.05.

## Discussion

A previous study identified the primary causes of the low SDL implant success rate as inadequate support, which impairs lead fixation, unclear anatomy of the atrial septum and foramen ovale, suboptimal pacing parameters, and a high risk of atrial lead dislodgement.^4^ The suggestion has been made that the atrial septal wall is thicker at the corner of the right atrial septum than at the right atrial appendage. The high right atrial septum, where the atrial myocardium is approximately 4 mm thick, provides deep and stable lead fixation.^16^

Currently, using the lumenless 3830 lead with the delivery sheath method allows for convenient, flexible, and precise positioning. This technique primarily combines atrial wave potential mapping and the assessment of the COI, facilitating rapid localization, significantly reducing the number of attempts and the implantation time. By employing this optimized implantation method and avoiding perforation complications, a high success rate and long-term safety of RASp with LLLs were achieved. The implant success rate of RASp is comparable to that of traditional RAAp (98.8% vs 97.6%, *P* > 0.05). With a mean follow-up of 36.4 ± 20.5 months, stable pacing parameters were maintained without serious adverse complications, demonstrating excellent long-term safety and stability.

For the RASp cases, lead positions were classified as either high or low RAS based on fluoroscopy and pacing atrial wave analysis. The number of cases of high RASp was 154, and the number of cases of low RASp was 6. In the high RASp group, some leads were implanted in the Bachmann’s bundle area. A total of 39 patients (25.3%) met the 3 ECG criteria for Bachmann’s bundle area pacing (BBAp).^14^^,^^17^ In the 39 cases of BBAp, no new-onset AF events occurred. However, electrophysiological mapping was not performed intraoperatively. Some studies suggest that BBAp may offer advantages in maintaining atrial synchrony and improving diastolic dysfunction.^9^^,^^17^ A recent short-term study on BBAp confirmed it as a feasible and safe alternative to standard RAAp. However, the study had a small sample and a short-term follow-up period, indicating the need for larger multicentre studies with longer follow-up periods.^14^

### Operational techniques for RASp with a high success rate

In this study, using LLLs with a delivery sheath for RASp resulted in a high success rate for atrial septal implantation without serious complications such as lead dislodgement, pericardial effusion, or cardiac perforation. During the procedure, the lead was directed to be perpendicular to the spine and septum in the LAO view and confirmed to be at the posterior septum, away from the aortic sinus, using the right anterior oblique view (Fig. 2, A and B).

The use of LLLs led to significantly shorter lead-implantation times for RASp compared to SDL for RAAp, averaging 2.5 ± 1.9 minutes vs 10.3 ± 2.9 minutes (*P* < 0.05). In the RASp group, 135 patients (84.4%) had successful lead fixation on the first attempt, whereas in the RAAp group, 108 patients (66.3%) required multiple attempts due to the challenges of SDL positioning. Without a delivery sheath, any slight displacement after SDL unscrewing led to significant changes in pacing parameters, necessitating repeated fixation. Additional factors contributing to this improvement include the following:

1. The delivery sheath method can achieve flexible and stable positioning.
2. The method integrated fluoroscopy and electrophysiological mapping (especially atrial wave amplitude and the isotropic COI) to determine the initial lead positioning, reducing the number of attempts.
3. A clear 1:1 rebound when the lead was twisted, indicating successful fixation, was observed.
4. The operator's extensive experience, with over 10 years in atrial septal pacing with the delivery sheath and conduction system pacing.

Consequently, the total atrial lead fixation time was significantly shorter with the delivery sheath method, making it more suitable for patients with severe atrial cardiomyopathy.

### The clinical benefits of RASp

In this study, the postoperative incidence of AF events was significantly lower in the RASp group, compared to that in the RAAp group (6.7% vs 14.0%, *P* = 0.001), as well as the incidence of new-onset AF (1.8% vs 4.6%, *P* = 0.047). Previous studies have reported similar findings, indicating that RASp is more effective than RAAp in preventing AF.^18^^,^^19^ In this clinical study, all postoperative AF events in both groups were reduced, compared to baseline. Additionally, manual review of programmed data clarified AF events and reduced false-positive events. Our aim was to detect additional AF events with a highly sensitive criterion and to show the preoperative vs postoperative differences.

The lack of a significant difference in AF burden ≥ 40% between the groups could be due to the small sample of AF patients and the presence of both electrical and structural cardiac remodelling in patients with a high AF burden. Without interventions such as antiarrhythmic drugs or ablation, the differences in AF burden were minimal. Moreover, the ventricular % was low in both groups (14.1% vs 15.1%, *P* > 0.05), having a minimal impact on postoperative AF events.

The statistical analysis of the research data revealed that the RASp group exhibited a wider P-wave duration and greater P-wave dispersion during sinus rhythm. The difference between the 2 groups was not due to selective enrollment bias but reflected the true data. However, the significantly shorter postoperative P-wave duration in the RASp group may be related to the smaller difference in AF incidence.

During the echocardiographic evaluation at the 1-year follow-up, both groups showed significant reductions in LAD and LVEDD, compared to baseline (*P* < 0.05). This study demonstrated that RASp can improve cardiac function, NYHA functional class, and echocardiographic parameters, primarily due to heart rate restoration after pacing. Although diastolic function was not specifically assessed, RASp may enhance left ventricular filling and cardiac output by shortening inter-atrial conduction time and achieving atrial resynchronization, which prolongs the filling time from the left atrium to the left ventricle, improving left ventricular diastolic function.^9^^,^^20^ A prior study by Loon et al. suggested that RAAp, unlike RASp, may exacerbate diastolic dysfunction.^21^

Another potential clinical benefit of RASp is the reduced risk of atrial appendage or atrial free wall rupture during long-term lead extraction, which is beneficial when lead removal is required due to infection or malfunction.

### Limitations

This study was retrospective and was conducted at a single centre. The 3830 lead with the delivery sheath is not recommended for use in the RAA due to the risk of perforating the thin atrial wall. The coexistence of AF in patients with SSS involves a complex mechanism with multiple contributing factors, and no univariate adjustment or analysis was performed for the differences in AF incidence between the 2 groups. Additionally, the significantly shorter procedural time for LLLs, compared to SDLs, in this study was attributed largely to the operator's extensive implant experience, which may necessitate a learning curve for new operators. Extensive, multicentre, prospective clinical studies are necessary to further validate the clinical benefits of RASp, with particular attention to the emerging interests in BBAp.

### Conclusions

In this study, the implant success rate of RASp with LLLs was high and comparable to that of conventional RAAp with SDLs. However, the RASp procedure was quicker and allowed for precise lead placement. Long-term follow-up confirmed stable postoperative pacing parameters, with no instances of lead dislodgement, cardiac perforation, or other complications. Our findings suggest that the long-term safety of RASp has been validated. Additionally, RASp can reduce the incidence of postoperative AF events in patients with SSS, particularly new-onset AF. Therefore, RASp can be considered a viable alternative for physiological atrial pacing.

**Central Illustration:**
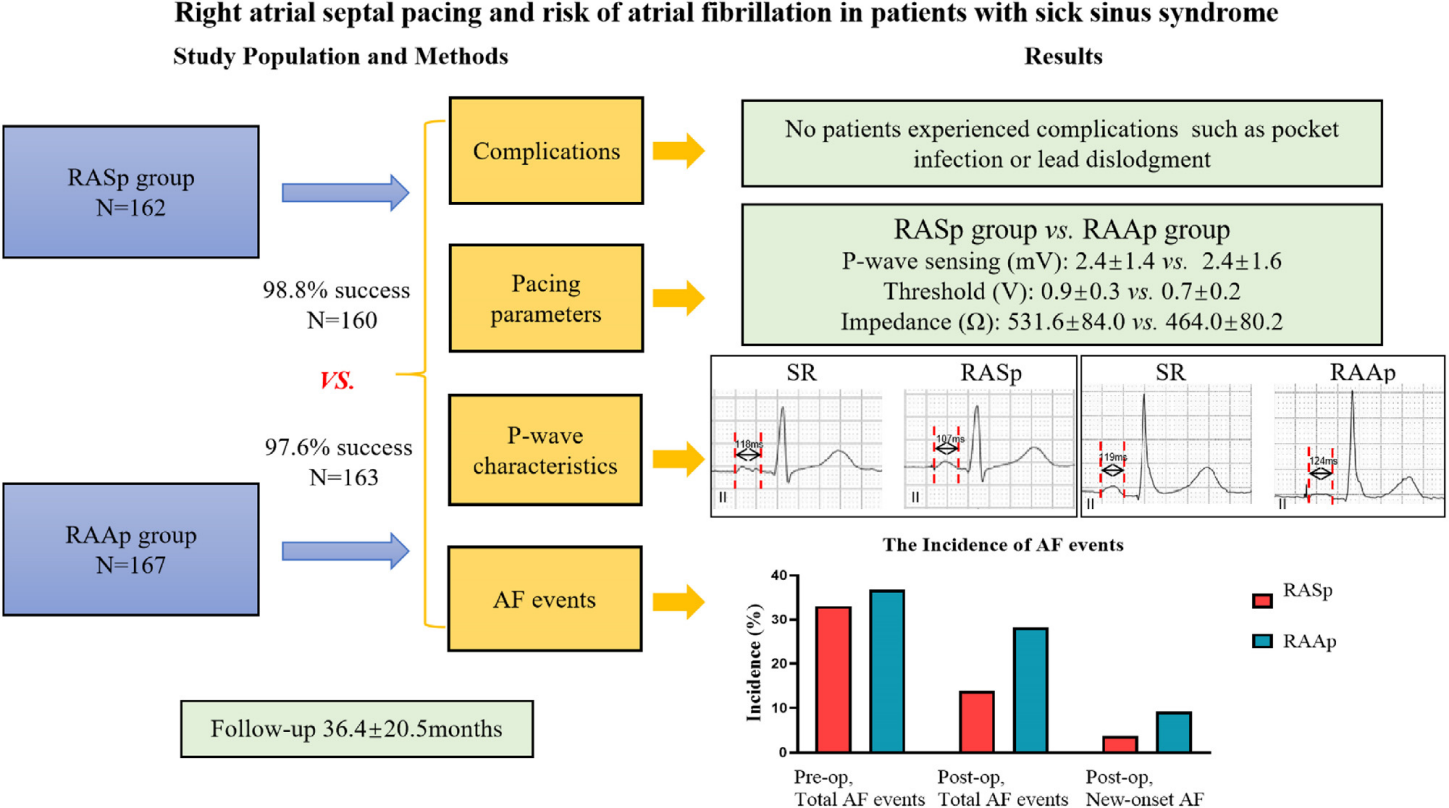
Right atrial septal pacing (RASp) with lumenless lead reduced the occurrence of atrial fibrillation (AF) in patients with sick sinus syndrome. The RASp with lumenless lead implantation was a safe and effective method. Post-op, postoperative; Pre-op, preoperative; RAAp, right atrial appendage pacing. SR, sinus rhythm.

## Data Availability

The data underlying this article will be shared on reasonable request to the corresponding author.
